# Public health round-up

**DOI:** 10.2471/BLT.21.010921

**Published:** 2021-09-01

**Improving emergency response**

WHO Academy learning programme creator, Nelson Olim, assists participants in a mass casualty management training session for the Emergency Unit team at Attikon University Hospital in Athens, Greece. Such activities will be core to training delivered under the INITIATE2 project launched by the United Nations World Food Programme and the World Health Organization on 19 July.

**Figure:**
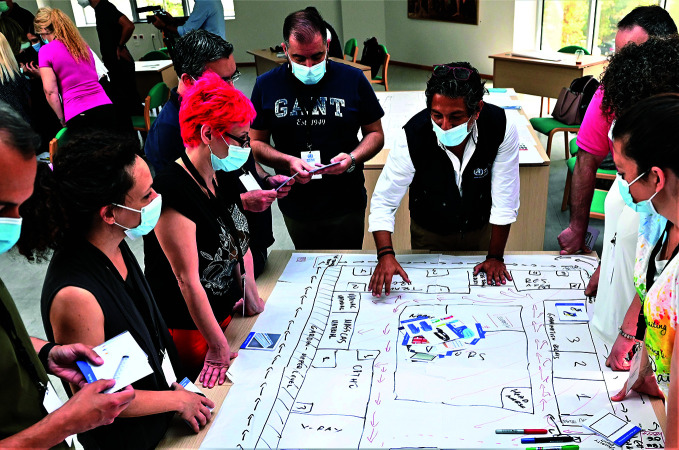
WHO/G. Lymperopoulos

## Afghanistan crisis

The World Health Organization (WHO) committed to staying in Afghanistan and delivering critical health services and called on all parties to respect and protect civilians, health workers, patients and health facilities. 

In a statement issued on 18 August, WHO reported that months of violence have taken a heavy toll on Afghanistan’s fragile health system, which had already been facing shortages in essential supplies amid the COVID-19 pandemic. 

Despite the ongoing insecurity in the country, on 17 August WHO dispatched to Wazir Akbar Khan Hospital in Kabul 33 units of different modules of trauma kits, enough to cover 500 surgical procedures for 500 trauma patients and 750 burn victims, and 10 basic medical kits enough to provide essential medicines for 10 000 people for 3 months. 

WHO has also provided Helmand regional hospital with basic medical supplies and medicines to support the needs of 6000 people for 3 months, and has donated medical supplies to three health partners to sustain critical work at their health facilities. WHO has also been working to provide training in mass casualty management, trauma care and mental health support. 

https://bit.ly/3mayG3J

## Ebola in Côte d’Ivoire

The Ministry of Health of Côte d’Ivoire reported that a patient with Ebola virus disease was admitted to a hospital in the commercial capital of Abidjan on 12 August, after arriving from Guinea by road. This is the first time since the 2014–2016 West Africa Ebola outbreak that an Ebola outbreak has occurred in a large city.

Guinea experienced a four-month long Ebola outbreak, which was declared over on 19 June 2021. As of 15 August, there was no indication that the new case was linked to the earlier outbreak but efforts, including gene sequencing, were ongoing to identify the strain.

“It is of immense concern that this outbreak has been declared in Abidjan, a metropolis of more than 4 million people,” said Dr Matshidiso Moeti, WHO Regional Director for Africa. “However, much of the world’s expertise in tackling Ebola is here on the continent and Côte d’Ivoire can tap into this experience and bring the response to full speed,” she said.

As of 15 August, WHO was helping to coordinate cross-border Ebola response activities and 5000 Ebola vaccines doses, which the Organization helped secure to fight the outbreak in Guinea, were being transferred to Côte d’Ivoire.

https://bit.ly/3m6jrsy

## Marburg in Guinea

A man was confirmed to have been infected with Marburg virus in Guinea. According to a report by the Ministry of Health of Guinea, the man began to have symptoms of Marburg virus disease on 25 July and on 1 August entered a small health facility near his village in Guéckédou Prefecture, Nzérékoré Region, south-western Guinea with fever, headache, fatigue, abdominal pain and gingival haemorrhage. The man received supportive care with rehydration, parenteral antibiotics and treatment to manage symptoms, but died in the community on 2 August.

The authorities were alerted the same day and an investigation team comprising national authorities and WHO experts, including epidemiologists and socio-anthropologists, were deployed to investigate the case and support the national health authorities in stepping up their emergency response.

https://bit.ly/2VQCuwo

## Haiti earthquake

A 7.2 magnitude earthquake shook Haiti on the morning of Saturday 14 August. A team of experts from the Pan American Health Organization’s (PAHO) office in Port au Prince was deployed to evaluate damage and coordinate an appropriate health response.

According to a 14 August report, the PAHO team was supporting coordination of the emergency response alongside Haiti’s Ministry of Health, other UN agencies and partners and also preparing Emergency Medical Teams, as well as medical supplies and other equipment.

As of 15 August, the Haiti Civil Protection Agency had reported that at least 1297 people had been killed. There were also reports of significant damage to health infrastructure, particularly in the south-west of the island.

https://bit.ly/3xSZCXY

## Emergency response to health crises

The UN World Food Programme (WFP) and WHO launched a project intended to promote knowledge sharing and skills transfer for improved emergency response to health crises.

Announced 19 July, the INITIATE2 project will bring together emergency actors, research and academic institutions, and international and national partners, and will develop standardized, innovative solutions such as disease-specific field facilities and kits and test these solutions in real-life scenarios.

The agencies will also train logistics and health responders, contributing to their capacity to respond in health crises, and will leverage the existing infrastructure of the UN Humanitarian Response Depot in Brindisi.

https://bit.ly/3jJPrzS

## Establishing SARS-CoV-2 origins

WHO outlined the series of studies that need to be undertaken to establish the origins of the severe acute respiratory syndrome coronavirus 2 (SARS-CoV-2) virus and is in discussions with Member States and experts on next steps.

In a statement released on 12 August, WHO called for the depoliticization of the SARS-CoV-2 origins discussion, arguing that the search for those origins is not and should not be an exercise in attributing blame or political point-scoring.

WHO’s priority is for scientists to build on the first phase of studies and to continue focusing efforts on all the origin hypotheses. Building on what has already been learned, the next series of studies would include a further examination of the raw data from the earliest cases and sera from potential early cases in 2019.

WHO also called for governments to work together on developing a common framework for future emerging pathogens of pandemic potential.

https://bit.ly/3sacyYo

## COVID-19 booster recommendations

WHO released an interim statement on the use of COVID-19 vaccine booster doses with the support of the Strategic Advisory Group of Experts on Immunization and its COVID-19 Vaccines Working Group.

WHO emphasized the importance of evidence-based decision-making and stated that, to date, the evidence is inconclusive regarding any widespread need for booster doses following primary vaccination series.

WHO went on to state that in light of ongoing global vaccine supply constraints, and the likelihood that the administration of booster doses will consume scarce supplies, for the time being the focus should remain on increasing global vaccination coverage with the primary series.

https://bit.ly/3xGcGzN

**Cover photo**

Seven-year-old girls studying at home during the COVID-19 lockdown in Gujarat, India, two of the many millions of children who have had their education disrupted by the COVID-19 pandemic.

**Figure:**
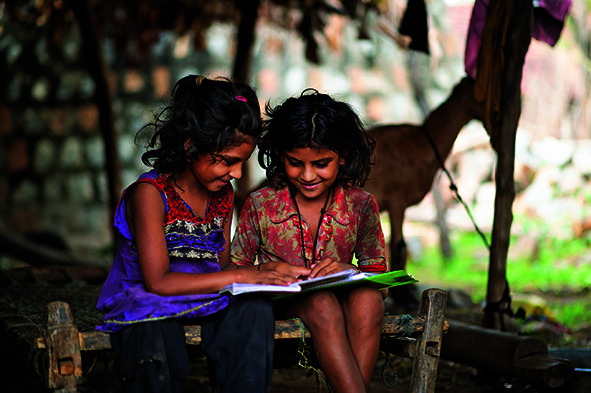
UNICEF/Vinay Panjwani

## Managing opioid overdose

Community management of opioid overdose may be highly effective in low- and middle-income countries. This is the main finding of a study undertaken in Kazakhstan, Kyrgyzstan, Tajikistan and Ukraine which began in 2016 and ended in 2020, the results of which were released on 6 August.

Participants most likely to witness an opioid overdose were trained to recognize one and respond using a kit to administer naloxone, a medication that reduces the effects of overdose if administered rapidly.

Some 14 263 people were trained as part of the four-country project. Overall, 31% (427 of 1388) of the participants followed up in a cohort study reported administering naloxone at an overdose that they witnessed and that the drug’s recipients survived in almost all cases.

This is the first time that the potential benefits of such an initiative in low- and middle-income settings have been evaluated. More studies are needed.

https://bit.ly/37GuQqS

## New Solidarity PLUS trial

WHO’s Solidarity PLUS trial will enrol hospitalized patients to test three new drugs in hospitalized COVID-19 patients. The therapies (artesunate, imatinib and infliximab) were selected by an independent expert panel for their potential in reducing the risk of death in hospitalized COVID-19 patients.

The Solidarity PLUS trial represents the largest global collaboration among WHO Member States, involves thousands of researchers in over 600 hospitals in 52 countries and makes it possible to assess multiple treatments at the same time using a single protocol.

“Finding more effective and accessible therapeutics for COVID-19 patients remains a critical need, and WHO is proud to lead this global effort,” said WHO Director-General Tedros Adhanom Ghebreyesus.

https://bit.ly/3yG2tF7

**Looking ahead**

6–12 September. Global week for action on NCDs. https://bit.ly/2VQka60

7–9 September. 5th international UV & skin cancer prevention conference. https://bit.ly/3qF7xWT

14–30 September. United Nations Food Systems Summit. https://bit.ly/3A8zYRD

